# The effect of routine intraoperative neuromonitoring on thyroidectomy complications: A propensity score-matched retrospective cohort study

**DOI:** 10.1097/MD.0000000000051068

**Published:** 2026-10-09

**Authors:** Huseyin Emre Arslan, Bercis Imge Ucar, Sezgin Zeren, Ali Cihat Yildirim, Mehmet Fatih Ekici, Ozlem Arik, Mustafa Cem Algin

**Affiliations:** a Department of General Surgery, Faculty of Medicine, Kutahya Health Sciences University, Kutahya, Turkey; b Department of Biostatistics, Faculty of Medicine, Kutahya Health Sciences University, Kutahya, Turkey; c Department of General Surgery, Eskisehir City Hospital, Eskisehir, Turkey.

**Keywords:** endocrine surgery, hypocalcemia, hypoparathyroidism, intraoperative neuromonitoring, recurrent laryngeal nerve injury, thyroidectomy

## Abstract

Intraoperative neuromonitoring (IONM) is commonly used during thyroid surgery to facilitate recurrent laryngeal nerve (RLN) identification and potentially reduce related postoperative complications. However, the efficacy of routine IONM in decreasing complication rates remains debated. In this study, we investigated the impact of IONM on postoperative complications after thyroidectomy. This retrospective single-center study included 214 thyroidectomy operations performed between 2021 and 2024. Patients were divided into 2 groups: Group 1 (n = 107) underwent surgery with routine IONM, and Group 2 (n = 107) without IONM, using only conventional direct visualization. Postoperative complications (RLN palsy, hypocalcemia, hypoparathyroidism, and incidental parathyroidectomy) were compared between groups. The primary outcome was any RLN palsy, analyzed at the nerve level. Prespecified propensity score matching and sensitivity analyses were also performed. Preoperative calcium levels were higher in Group 1 than in Group 2 (*P* = .002). Postoperative calcium and parathyroid hormone levels and the rates of hypocalcemia and hypoparathyroidism did not differ between the groups; the smallest *P* value across these outcomes was.04 before and.40 after adjustment for multiplicity. There were 198 nerves at risk in Group 1 and 204 in Group 2. Any RLN palsy occurred in 3/198 nerves (1.5%) in Group 1 and 2/204 (1.0%) in Group 2 (*P* = .68). Transient RLN palsy occurred in 3/198 (1.5%) and 1/204 (0.5%), respectively (*P* = .37), whereas permanent RLN palsy occurred in 0/198 (0%) and 1/204 (0.5%), respectively (*P* > .99). No difference in any RLN palsy was observed in the propensity score–matched cohort (*P* > .99), and comparable findings were observed in the pre specified sub cohort and sensitivity analyses. Routine IONM was not associated with a significant reduction in RLN palsy, hypocalcemia, or hypoparathyroidism compared with conventional direct visualization. The very small number of RLN events and the wide confidence intervals indicate limited statistical precision, and the study was not sufficiently powered to exclude clinically meaningful differences in the risk of RLN palsy.

## 1. Introduction

Thyroid diseases are a common and significant group of endocrine disorders, and thyroidectomies are the most frequently performed endocrine surgeries for treating various thyroid conditions. Standardized techniques and improved perioperative management have led to a significant decrease in overall mortality and morbidity associated with thyroidectomy in the 21st century. Today, thyroidectomies have a low complication rate, often below 5%, and can even be performed on a day-case basis in suitable cases. The most common complications of thyroidectomy include recurrent laryngeal nerve (RLN) injury, hypocalcemia, hypoparathyroidism, incidental parathyroidectomy (IP), and hematoma.^[1–3]^ To reduce these complications, the surgeon must carefully identify and preserve the RLN and parathyroid glands through meticulous dissection during surgery.^[4]^

Postoperative hypocalcemia remains the most common complication of thyroid surgery, with a reported prevalence ranging from 1.5% to 50%, depending on the definition of hypocalcemia. Following thyroid surgery, transient hypocalcemia occurs in 1.6% to 50% of patients, whereas permanent hypocalcemia occurs in 1.5% to 4% of cases.^[1–5]^ Symptoms related to hypocalcemia typically manifest within the first 24 to 48 hours postoperatively.^[6]^

Postoperative hypoparathyroidism can be classified as transient (0–1 month), prolonged (1–6 months), or permanent (>6 months). Transient hypoparathyroidism (19%–38%) is the second most common complication following total thyroidectomy (TT).^[5–7]^

RLN paralysis is one of the most serious complications following thyroidectomy. Intraoperative identification of the RLN is widely accepted as the gold standard to prevent nerve injury.^[8]^ Intraoperative neuromonitoring (IONM) is a powerful adjunct to conventional direct visualization techniques to monitor neural structures. IONM during thyroidectomy has gained popularity because it allows the surgeon to verify the functional integrity of the RLN during surgery and assists in identification of the RLN prior to visual confirmation, especially in high-risk surgery, such as that for malignant disease.^[9]^

However, the role and benefits of IONM in preventing nerve injury remain controversial. Although RLN injury is relatively rare following thyroidectomy, with reported rates ranging from 1.25% to 3.9%, current research has demonstrated that routine use of IONM does not reduce the incidence of RLN injury and is not cost-effective.^[10]^ Despite the absence of a universal consensus on the use of IONM, the American Academy of Otolaryngology-Head and Neck Surgery recommends its use during thyroid surgery to preserve voice quality. Both the German Association of Endocrine Surgeons and the International Neural Monitoring Study Group advocate the use of IONM in all thyroid surgeries. The American Head and Neck Society has also published a consensus statement supporting the routine use of nerve monitoring during thyroidectomy.^[11]^

In this study, we compared the postoperative complication rates between patients who underwent thyroidectomy with routine use of IONM and those who underwent thyroidectomy with conventional direct visualization alone.

## 2. Materials and methods

This retrospective study included all patients who underwent thyroid surgery between November 2021 and February 2024 at the Department of General Surgery, Kutahya Health Sciences University (Kutahya, Turkey). The study was approved by the Non-Interventional Clinical Research Ethics Committee of Kutahya Health Sciences University (Ethics committee decision no: 2024/03-38). The study was performed in accordance with the Declaration of Helsinki, and no personal data of any patient were disclosed during the study. Informed consent was obtained from all individual participants included in the study. Written informed consent was secured prior to participation in accordance with institutional and national ethical standards.

The study included male and female patients aged 19 to 84 years who were diagnosed with multinodular or nodular goiter, Graves’ disease, or thyroid cancer, and presented with American Society of Anesthesiologists scores of I, II, or III. Patients were excluded if they met one or more of the following criteria: pregnancy; age below 18 years; history of preoperative laryngeal surgery; preoperative diagnosis of vocal cord pathology; history of radiotherapy to the neck region; preoperative diagnosis of parathyroid pathology; not normocalcemic preoperatively; or stage 3 or higher chronic kidney disease.

All patients underwent routine pre- and postoperative vocal cord examination by an experienced otolaryngologist using flexible fiberoptic laryngoscopy in the Department of Otorhinolaryngology. All surgeries were performed in a single clinic by or under the supervision of general surgeons experienced in thyroid surgery. Age, sex, preoperative calcium (mg/dL) and albumin (g/dL) levels, and ear, nose, and throat (ENT) consultation notes were retrospectively collected from preoperative hospital records. The surgical procedure was categorized as bilateral TT, near-TT, completion TT, or lobectomy. The extent of resection was additionally classified as total or near-total/subtotal. A bilateral procedure was defined as an operation involving both thyroid lobes. Completion TT was recorded as reoperation for completion thyroidectomy. Histopathological diagnoses were recorded according to the final pathology report and subsequently classified as benign or malignant for the analysis. Hashimoto thyroiditis and chronic lymphocytic thyroiditis were treated as a single diagnostic category, as they denote the same histopathological entity.

The type of surgical procedure and the use of IONM were recorded from the operative reports. Complications, pathology results, calcium and parathyroid hormone (PTH) levels at the 8^th^ hour and 6^th^ month postoperatively, and vocal cord function data from postoperative ENT consultations were recorded.

Records of 223 patients who underwent thyroid surgery between 2021 and 2024 were retrospectively reviewed. Six patients with missing data and 1 patient with preoperative hypoparathyroidism were excluded. In addition, lateral lymph node dissection (n = 1) and thymic tissue on the final pathology report (n = 1) were further exclusion criteria. Finally, 214 patients with complete preoperative, intraoperative, and postoperative records were included in the study (see Figure S1, Supplemental Digital Content 1, which shows the patient selection flowchart).

The patients were divided into 2 groups. As routine use of IONM was first implemented in November 2023, 107 patients who underwent surgery with IONM constituted Group 1, and 107 patients who underwent surgery with the conventional direct visualization technique (prior to IONM implementation) constituted Group 2.

Initial calcium, albumin, and PTH levels were measured at the 8^th^ postoperative hour for all patients. Calcium levels were corrected based on the albumin levels using the following formula^[12]^:


$$\begin{array}{cc} Corrected\;total\;blood\;calcium\left(mg/dL\right)=0.8\\ & \times \left[normal\;albumin\;value\;4\;g/dL-patients\;albumin\left(g/dL\right)\right]\\ & +total\;blood\;calcium \end{array}$$

Patients with corrected calcium levels below 8.8 mg/dL (reference range: 8.8–10.2 mg/dL), regardless of the presence of symptoms, were considered hypocalcemic. Patients with PTH levels below 12 ng/L (reference range: 12–65 ng/L) were classified as having hypoparathyroidism. For the analysis, transient and permanent outcomes were treated as mutually exclusive categories, with transient outcomes representing abnormalities present at the 8th postoperative hour that had resolved by the 6-month assessment, and permanent outcomes representing abnormalities persisting at 6 months. Patients whose corrected calcium levels remained below 8.8 mg/dL and whose PTH levels remained below 12 ng/L at the 6-month follow-up were classified as having permanent hypocalcemia and hypoparathyroidism, and those with corrected calcium and PTH levels that were normalized at the 6-month follow-up were classified as having transient hypocalcemia and hypoparathyroidism.

Patients with symptomatic hypocalcemia were treated with oral calcium and vitamin D supplements, as well as intravenous calcium gluconate. All patients with hypocalcemia and/or hypoparathyroidism were referred to the endocrinology clinic, and those with permanent conditions were followed up in the endocrinology outpatient setting.

When normal parathyroid tissue was identified in pathology specimens, the corresponding patients were evaluated for IP, and the number and anatomical location of the glands were recorded to assess their relationship with hypocalcemia. IP was defined as the identification of normal parathyroid tissue in the postoperative pathology specimen. Postoperative hematoma requiring reoperation, bilateral vocal cord paralysis, admission to the intensive care unit for a surgical complication, and in-hospital mortality were also recorded from the hospital records and are reported descriptively.

Patients were scheduled for an outpatient clinic visit in the first postoperative week for surgical site evaluation. Patients with dyspnea or respiratory distress underwent immediate ENT evaluation, whereas asymptomatic patients underwent vocal cord and larynx examination on postoperative day 2. Patients with vocal cord paralysis underwent follow-up evaluations at 1, 2, 3, and 6 months postoperatively. Those whose vocal cord movements did not return to normal within 6 months were classified as having permanent RLN paralysis, whereas those whose vocal cord movements (as determined using flexible fiber optic laryngoscopy) returned to normal and whose symptoms improved during the follow-up were classified as having transient RLN paralysis.

### 2.1. Surgical Technique

All patients underwent surgery under general anesthesia with endotracheal intubation. In both groups, sevo flurane was used as the primary anesthetic agent. Full-dose rocuronium was used only in patients operated on with the conventional direct visualization technique (Group 2), whereas low-dose rocuronium and remifentanil were used in the IONM group.

Patients were positioned in the semi-sitting position and operated on via a classical Kocher incision. Subcutaneous tissue and platysma muscle were dissected. Strap muscles were longitudinally dissected and retracted laterally using retractors. First, the middle thyroid vein was sealed using an energy device, followed by dissection of the upper pole. In all patients, the upper poles were ligated with 2/0 Vicryl sutures.

In all cases, the RLN was dissected and visualized in the tracheoesophageal groove, while the parathyroid glands were identified and preserved. Finally, the ligamentous structures between the thyroid gland and trachea were carefully dissected to complete the hemithyroidectomy. The same procedure was applied to the contralateral lobe to complete TT. In cases of near-TT, 1 thyroid lobe was completely removed, while a small portion of the other lobe, typically ≤1 cm, was preserved. The RLN was fully visualized during this procedure. A closed-suction Hemovac drain was placed in all patients for postoperative monitoring.

In Group 1, a nerve monitoring device (Dr Langer Medical, Germany) was used to monitor the functional integrity of the nerves during surgery. During intubation, the anesthesiologist placed a spiral electromyography (EMG) endotracheal tube such that the electrode surfaces contacted the vocal cords directly. Neuro monitoring was initiated after full recovery from the neuromuscular blockade. Repetitive RLN stimulations (1.5 mA) were performed using a disposable probe. Adequate stimulation was defined as both an audible alarm from the monitoring system and a detectable EMG waveform (≥100 µV for RLN). After completion of the 4-step EMG recording protocol, results were interpreted according to the guidelines of the International Neural Monitoring Study Group.^[13]^ EMG responses ≥100 µV in response to vagus nerve or RLN stimulation were defined as positive. An EMG amplitude <100 µV at the end of surgery was considered a complete loss of signal. In Group 2, the RLN was identified using conventional direct visualization only.

### 2.2. Statistical analysis

All analyses were confined to the analysis cohort of 214 operations. The dataset contained no missing values, and no imputation was therefore required.

Continuous variables were assessed for normality within each study group using the Lilliefors-corrected Kolmogorov-Smirnov test together with skewness, kurtness, and quantile-quantile inspection (see Figure, S2, Supplemental Digital Content 2, which shows the normality assessment of the perioperative laboratory variables). Age and the estimated propensity score satisfied normality in both groups and are reported as mean ± standard deviation; preoperative, 8th-hour, and 6th-month calcium and 8th-hour and 6th-month PTH concentrations departed from normality in at least 1 group and are reported as median with interquartile range (IQR). Categorical variables are reported as counts and percentages.

Age was compared by independent-samples *t* test after homogeneity of variance had been verified by the median-centered Levene test, with Cohen’s d and its 95% confidence interval (CI) as the effect size. The 5 laboratory variables were compared by Mann–Whitney *U* test, and the Hodges-Lehmann estimator with its distribution-free interval derived from the order statistics of all pairwise differences served as the corresponding effect estimate. Categorical variables were compared by Pearson chi-square test where all expected cell counts reached 5, by Fisher exact test in 2 × 2 tables that did not, and by the Fisher-Freeman-Halton exact test based on 20,000 Monte Carlo permutations under a fixed random seed for the 4-category procedure variable and the ten-category histopathological diagnosis variable. Effect sizes for categorical comparisons were expressed as odds ratios for 2-level variables and as Cramér’s V with percentile bootstrap intervals from 5000 resamples for variables with more than 2 levels. For binary outcomes, the risk difference and its Newcombe hybrid score interval were reported.^[14]^

The primary outcome, any RLN palsy, was analyzed at the nerve level, with 2 nerves at risk counted for bilateral and one for unilateral procedures. Because the side of injury was not documented in any affected patient, one affected nerve was attributed to each affected patient, and within-patient clustering, therefore, could not be quantified. Fisher exact test performed comparative testing at the nerve level since the expected cell count fell below 5 in every comparison. Patient-level counts are presented descriptively and were not tested. No adjusted estimate, model, or subgroup analysis was undertaken for permanent RLN palsy, as a single event occurred across the entire cohort.

A propensity score for receipt of IONM was estimated by logistic regression on age, male sex, malignant pathology, and bilateral procedure, giving 26.8 events per variable.^[15]^ For this analysis, malignant pathology was defined according to the final postoperative histopathological classification as a malignant thyroid neoplasm. Preoperative calcium was excluded because it showed no association with the primary outcome. Matching was performed 1:1 without replacement within a caliper of 0.2 standard deviation of the logit of the propensity score, specified a priori, and pairs were formed by linear sum assignment so that the total within-pair distance on the logit scale was minimized;^[16]^ this deterministic approach was adopted because greedy nearest-neighbor matching is sensitive to the order in which treated units are processed. Balance was assessed by absolute standardized mean differences, with a threshold of 0.10 defined as adequate.^[17]^ The matched cohort was compared by exact McNemar test, the number of discordant pairs being below the prespecified threshold of 25, and the paired risk difference was accompanied by a Tango score interval.^[18]^

Two secondary families were specified before outcome data were examined. Eleven biochemical and parathyroid outcomes were adjusted for multiplicity by the Benjamini-Hochberg false discovery rate procedure. Preoperative calcium and the composite definition of postoperative hypoparathyroidism were excluded from this family, the former because it served a descriptive purpose only and the latter because it recombines outcomes already tested. Postoperative hypoparathyroidism in this composite analysis was defined as the coexistence of hypocalcemia and PTH < 12 ng/L. Sub cohort and sensitivity analyses of the primary outcome, comprising the bilateral TT sub cohort, patients whose recorded preoperative calcium was within the reference range, and 1 record per patient, constituted a separate exploratory family and were reported without adjustment.^[19]^

All analyses were performed in Python 3.12.3 (Python Software Foundation, Wilmington, DE) with NumPy 2.4.4, pandas 3.0.2, SciPy 1.17.1, and stats models 0.14.6; figures were produced with Matplotlib 3.10.8. Newcombe hybrid score intervals, Tango score intervals, Hodges-Lehmann estimators, and standardized mean differences were implemented directly from their published formulae. A 2-sided *P* value of .05 or less was considered statistically significant.

## 3. Results

The groups were comparable in age, sex, surgical procedure, extent of resection, laterality, reoperation status, histopathological diagnosis, and final pathology; the smallest *P* value across these comparisons was .19 (Table 1).

**Table 1:** Baseline demographic, surgical and histopathological characteristics of patients undergoing thyroidectomy with and without routine intraoperative neuromonitoring.

Characteristics	Total (*n* = 214)	IONM (*n* = 107)	Non-IONM (*n* = 107)	Effect size (95% CI)	*P*
Demographics					
Age (yr)	49.24 ± 13.91	50.06 ± 14.65	48.43 ± 13.14	d = 0.12 (−0.15–0.39)	.39
Sex					
Female	167 (78.0)	81 (75.7)	86 (80.4)	OR = 1.31 (0.69–2.52)	.41
Male	47 (22.0)	26 (24.3)	21 (19.6)		
Surgical procedure*					
Bilateral total thyroidectomy	165 (77.1)	84 (78.5)	81 (75.7)	V = 0.150 (0.067–0.291)	.20
Near-total thyroidectomy	22 (10.3)	7 (6.5)	15 (14.0)		
Completion total thyroidectomy	5 (2.3)	2 (1.9)	3 (2.8)		
Lobectomy	22 (10.3)	14 (13.1)	8 (7.5)		
Extent of resection					
Total	190 (88.8)	98 (91.6)	92 (86.0)	OR = 0.56 (0.24–1.35)	.19
Near-total or subtotal	24 (11.2)	9 (8.4)	15 (14.0)		
Bilateral procedure, *yes*	188 (87.9)	91 (85.0)	97 (90.7)	OR = 0.59 (0.25–1.36)	.21
Nerves at risk (n)	402	198	204	–	–
Reoperation (completion thyroidectomy), yes*	5 (2.3)	2 (1.9)	3 (2.8)	OR = 0.66 (0.11–4.03)	>.99
Histopathological diagnosis*					
Adenomatous hyperplasia	30 (14.0)	16 (15.0)	14 (13.1)	V = 0.215 (0.166–0.347)	.32
Follicular adenoma	6 (2.8)	1 (0.9)	5 (4.7)		
Follicular carcinoma	1 (0.5)	1 (0.9)	0 (0.0)		
Hashimoto thyroiditis	23 (10.7)	12 (11.2)	11 (10.3)		
Hürthle cell adenoma	2 (0.9)	1 (0.9)	1 (0.9)		
Hürthle cell carcinoma	2 (0.9)	0 (0.0)	2 (1.9)		
Medullary carcinoma	1 (0.5)	0 (0.0)	1 (0.9)		
NIFTP	1 (0.5)	0 (0.0)	1 (0.9)		
Nodular hyperplasia	88 (41.1)	41 (38.3)	47 (43.9)		
Papillary carcinoma	60 (28.0)	35 (32.7)	25 (23.4)		
Final pathology					
Benign	150 (70.1)	71 (66.4)	79 (73.8)	OR = 1.43 (0.79–2.58)	.23
Malignant	64 (29.9)	36 (33.6)	28 (26.2)		

Data are presented as mean ± SD or n (%), as appropriate. Bold *P* values indicate statistical significance (*P* ≤ .05). No comparison in this table reached statistical significance. Hashimoto thyroiditis and chronic lymphocytic thyroiditis are reported as a single category, as they denote the same histopathological entity; they appeared as 2 separate categories in the previously submitted version. Age was normally distributed in both groups and variances were homogeneous (Levene *P* = .12); it was therefore compared by independent-samples *t* test.

* Fisher exact test for 2 × 2 tables with an expected cell count below 5, and Fisher–Freeman–Halton exact test (Monte Carlo, 20 000 permutations) for larger tables in which more than 20% of cells had an expected count below 5. Variables without a symbol were compared by Pearson chi-square test. Effect sizes are the odds ratio with the intraoperative neuromonitoring group as the exposed group for binary variables, and Cramér’s V for variables with more than 2 categories; confidence intervals for Cramér’s V were obtained by bootstrap resampling (5000 replicates). Nerves at risk are reported as the total number of nerves rather than as a per-patient statistic and were neither tested nor summarized by an effect size, since this quantity is determined entirely by the laterality of the procedure. Percentages for the total column are calculated over 214 patients and for the group columns over 107 patients each.

CI = confidence interval, IONM = intraoperative neuromonitoring, NIFTP = noninvasive follicular thyroid neoplasm with papillary-like nuclear features, OR = odds ratio, SD = standard deviation, V = Cramér’s V.

Perioperative calcium and PTH levels and their related biochemical outcomes are detailed in Table 2. Preoperative calcium was higher in Group 1 (median 9.70 vs 9.50 mg/dL; median difference 0.20, 95% CI: 0.10–0.30; *P* = .002). Median postoperative 8th-hour calcium was 8.90 mg/dL (IQR 8.80–9.03) in Group 1 and 9.10 mg/dL (IQR 8.05–9.40) in Group 2, a median difference of −0.16 mg/dL (95% CI: −0.30–0.00; adjusted *P* = .40). None of the remaining biochemical measurements differed between the groups, nor did the proportions of patients with transient or permanent hypocalcemia, with early, transient, or permanent hypoparathyroidism, or with postoperative hypoparathyroidism; the smallest adjusted *P* value across the family of eleven secondary outcomes was .40 (Table 2).

**Table 2:** Perioperative calcium and parathyroid hormone levels and biochemical outcomes according to intraoperative neuromonitoring use.

Variables	IONM (*n* = 107)	Non-IONM (*n* = 107)	Effect estimate (95% CI)‡	*P*	*q* §
Calcium					
Preoperative calcium (mg/dL)*	9.70 (9.40–9.90)	9.50 (9.30–9.80)	0.20 (0.10–0.30)	.002	—
Postoperative 8th-hour calcium (mg/dL)*	8.90 (8.80–9.03)	9.10 (8.05–9.40)	−0.16 (−0.30–0.00)	.04	.40
Hypocalcaemia (<8.8 mg/dL), *yes*	24 (22.4)	33 (30.8)	−0.084 (−0.200–0.034)	.16	.60
Postoperative 6th-month calcium (mg/dL)*	9.40 (9.10–9.70)	9.40 (9.10–9.65)	0.00 (−0.10–0.10)	.81	.92
Transient hypocalcemia, *yes*	19 (17.8)	30 (28.0)	−0.103 (−0.213–0.010)	.07	.40
Permanent hypocalcemia, *yes*†	5 (4.7)	3 (2.8)	0.019 (−0.039–0.080)	.72	.92
Parathyroid hormone					
Postoperative 8th-h PTH (ng/L)*	46.00 (22.00–66.00)	45.60 (23.00–62.00)	2.00 (−6.00–11.00)	.62	.92
Early hypoparathyroidism (<12 ng/L), *yes*	8 (7.5)	9 (8.4)	−0.009 (−0.087–0.067)	.80	.92
Postoperative 6th-mo PTH (ng/L)*	48.30 (33.50–68.00)	44.90 (32.00–68.45)	0.00 (−7.00–6.00)	.93	.93
Transient hypoparathyroidism, *yes*	6 (5.6)	9 (8.4)	−0.028 (−0.102–0.044)	.42	.92
Permanent hypoparathyroidism, *yes*†	2 (1.9)	0 (0.0)	0.019 (−0.019–0.066)	.50	.92
Postoperative hypoparathyroidism (hypocalcemia with PTH < 12 ng/L), *yes*	8 (7.5)	7 (6.5)	0.009 (−0.064–0.083)	.79	–
Incidental parathyroidectomy, *yes*	13 (12.1)	12 (11.2)	0.009 (−0.079–0.098)	.83	.92

Data are presented as median (interquartile range) or n (%), as appropriate. Bold *P* values indicate statistical significance (*P* ≤ .05). Transient outcomes denote biochemical abnormality at the 8th postoperative hour that had resolved by the 6th month; permanent outcomes denote abnormality persisting at the 6th month. Categories are mutually exclusive. All continuous variables departed from normality in at least 1 group and were therefore compared by the Mann–Whitney *U* test (*); the corresponding effect estimate is the Hodges-Lehmann median difference. Binary outcomes were compared by Pearson chi-square test, or by Fisher exact test (†) where an expected cell count was below 5; the corresponding effect estimate is the risk difference. ‡ Hodges-Lehmann median difference for continuous variables; risk difference (Newcombe hybrid score interval) for binary variables, expressed as the neuro monitoring group minus the non-neuro monitoring group. § Benjamini-Hochberg false discovery rate–adjusted *P* value across the a priori locked family of *k* = 11 secondary outcomes. Preoperative calcium is presented for descriptive purposes only; it was not associated with the primary outcome and was therefore included neither in the propensity score model nor in this family. The composite definition of postoperative hypoparathyroidism combines the 2 component outcomes already reported above and is likewise excluded from the family to avoid counting the same patients a third time.

CI = confidence interval, IONM = intraoperative neuromonitoring, PTH = parathyroid hormone.

There were 198 nerves at risk in Group 1 and 204 in Group 2. Any RLN palsy was recorded in 3 of 198 nerves at risk (1.5%) in Group 1 and 2 of 204 (1.0%) in Group 2, a risk difference of 0.005 (95% CI: −0.022 to 0.035; *P* = .68). Transient palsy occurred in 3 of 198 (1.5%) and 1 of 204 (0.5%) nerves (risk difference 0.010, 95% CI: −0.014–0.039; *P* = .37), and permanent palsy in none of 198 and 1 of 204 (0.5%) (risk difference −0.005, 95% CI: −0.027–0.015; *P* > .99). At the patient level, palsy of any type was recorded in 3 patients (2.8%) and 2 patients (1.9%), respectively; patient-level counts were not tested (Table 3).

**Table 3:** Primary outcome: recurrent laryngeal nerve palsy analysed at the nerve level and at the patient level.

Outcomes	IONM	Non-IONM	Risk difference (95% CI)*	*P*
Nerve-level analysis (nerves at risk: IONM 198, non-IONM 204)				
Transient recurrent laryngeal nerve palsy	3/198 (1.5)	1/204 (0.5)	0.010 (−0.014–0.039)	.37
Permanent recurrent laryngeal nerve palsy	0/198 (0.0)	1/204 (0.5)	−0.005 (−0.027–0.015)	>.99
Any recurrent laryngeal nerve palsy	3/198 (1.5)	2/204 (1.0)	0.005 (−0.022–0.035)	.68
Patient-level analysis, descriptive only (IONM n = 107, non-IONM n = 107)				
Transient recurrent laryngeal nerve palsy	3/107 (2.8)	1/107 (0.9)	–	–
Permanent recurrent laryngeal nerve palsy	0/107 (0.0)	1/107 (0.9)	–	–
Any recurrent laryngeal nerve palsy	3/107 (2.8)	2/107 (1.9)	–	–

Data are presented as n/N (%). Bold *p*-values indicate statistical significance (*P* ≤ .05); no comparison in this table reached statistical significance. Nerves at risk were counted as two for bilateral procedures and one for unilateral procedures, giving 402 nerves at risk in 214 operations. All comparative testing was performed at the nerve level; patient-level counts are presented for descriptive purposes and for comparison with the previously submitted version, and are not tested. The side of nerve injury was not documented in any of the five affected patients. Nerve-level analyses were therefore performed by attributing one affected nerve to each affected patient over the number of nerves at risk, and within-patient clustering could not be quantified. All 5 events occurred in patients who underwent bilateral procedures; the nerve-level denominator should accordingly be interpreted as an exposure denominator rather than as a set of independently observed nerves. No adjusted effect estimate, model or subgroup analysis was performed for permanent recurrent laryngeal nerve palsy because only 1 event occurred in the entire cohort; the risk difference reported for that row is unadjusted and descriptive. Two patients were excluded from the analysis cohort a priori on clinical grounds, one who underwent central and lateral neck dissection and one whose specimen contained only thymic tissue. Statistical tests: Fisher exact test, applied to all three nerve-level comparisons because the expected cell count was below 5 in every case.

CI = confidence interval, IONM = intraoperative neuromonitoring.

* Newcombe hybrid score interval, expressed as the neuro monitoring group minus the non-neuro monitoring group.

Among the 3 patients with transient RLN palsy in Group 1, vocal cord mobility and clinical symptoms recovered at the 3-month follow-up in 2 patients and at the 6-month follow-up in 1. In Group 2, 1 patient recovered at the 6-month follow-up and was therefore classified as having transient palsy, whereas 1 patient with persistent vocal cord immobility and symptoms at the 6-month follow-up was classified as having permanent palsy and did not require vocal cord surgery.

No instance of hematoma requiring reoperation, bilateral vocal cord paralysis, postoperative surgical complications leading to intensive care admission, or mortality was found in either group.

Given the observed number of nerves at risk and a baseline risk of 0.98% in Group 2, the smallest risk in Group 1 detectable with 80% power was 6.00%, corresponding to an absolute difference of 5.0 percentage points and a risk ratio of 6.1; at 90% power, the detectable risk was 7.04%. In the opposite direction, the power to detect the complete elimination of nerve palsy was 0.016. The 95% CI obtained for the risk difference spanned 5.6 percentage points (Tables 4 and 5).

**Table 4:** Minimum detectable difference in the risk of recurrent laryngeal nerve palsy at the nerve level, given the observed sample size.

Analysis set and target power	Nerves at risk, IONM/ non-IONM	Baseline risk (%)	Detectable risk in the IONM group (%)	Absolute difference (percentage points)	Risk ratio
Primary analysis (full analysis cohort)	198/ 204	0.98	–	–	–
80% power			6.00	5.0	6.1
90% power			7.04	6.1	7.2
Propensity score–matched cohort	185/ 185	1.08	–	–	–
80% power			7.01	5.9	6.5
90% power			8.14	7.1	7.5

Values are the smallest risk in the neuro monitoring group that the study could have detected with the stated power, holding the observed number of nerves at risk and the observed baseline risk in the comparison group fixed. Calculations use the 2-sided Fisher exact test at α = .05, with power obtained unconditionally by summing the binomial probabilities of all tables falling in the rejection region; the truncation used in this summation retained the entire probability mass in every analysis set. Baseline risk is the observed risk of any recurrent laryngeal nerve palsy in the non-neuro monitoring group of the corresponding analysis set. Values obtained under the normal approximation were 0.4 to 1.9 percentage points less conservative than the exact values in every row and are not tabulated. This is a design sensitivity analysis and not a post hoc power calculation; the observed effect estimate is not used in its derivation. In the opposite direction the baseline risk of 0.98% sets an upper bound on any reduction, and the power to detect the complete elimination of nerve palsy was.016 in the primary analysis and.016 in the matched cohort.

IONM = intraoperative neuromonitoring.

**Table 5:** Achieved precision for the primary outcome in the principal analysis sets.

Analysis set	IONM, n/N (%)	Non-IONM, n/N (%)	Risk difference (95% CI)	Width of the 95% CI (percentage points)
Primary analysis (full analysis cohort)	3/198 (1.5)	2/204 (1.0)	0.005 (−0.022–0.035)	5.6
Propensity score–matched cohort	3/185 (1.6)	2/185 (1.1)	0.005 (−0.024–0.037)	6.1

Confidence intervals are Newcombe hybrid score intervals for the difference between two independent proportions, expressed as the neuro monitoring group minus the non-neuro monitoring group. Width is the arithmetic distance between the lower and upper limits and is reported as the measure of the precision the study actually achieved for the primary outcome. For the matched cohort the interval treats the 2 groups as independent and is therefore approximate; the corresponding paired estimate at the patient level was a risk difference of 0.010 with a Tango score interval of −0.046 to 0.070.

CI = confidence interval, IONM = intraoperative neuromonitoring.

Before matching, absolute standardized mean differences were 0.117 for age, 0.113 for male sex, 0.164 for malignant pathology, 0.172 for bilateral procedure, and 0.320 for the propensity score itself. Ninety-seven of the 107 patients in each group were matched, leaving 10 patients per group unmatched. After matching, every standardized mean difference fell below the pre specified threshold of 0.10, reaching 0.000 for bilateral procedure and 0.060 for the propensity score (Table 6, Fig. 1). The propensity score ranged from 0.358 to 0.759 in Group 1 and from 0.343 to 0.698 in Group 2 before matching, and from 0.358 to 0.679 and from 0.355 to 0.680 after matching; unmatched patients lay at the upper end of the distribution in Group 1 and at the lower end in Group 2 (Fig. 2). Preoperative calcium, which was not entered into the model, had a standardized mean difference of 0.315 before and 0.307 after matching.

**Table 6:** Covariate balance before and after 1:1 propensity score matching of neuro monitoring and non-neuro monitoring groups.

Covariates	Before: IONM (n = 107)	Before: non-IONM (n = 107)	SMD before	After: IONM (n = 97)	After: non-IONM (n = 97)	SMD after
Age (yr)	50.06 ± 14.65	48.43 ± 13.14	0.117	49.48 ± 14.52	49.65 ± 13.01	−0.012
Male sex, *yes*	26 (24.3)	21 (19.6)	0.113	20 (20.6)	21 (21.6)	−0.025
Malignant pathology, *yes*	36 (33.6)	28 (26.2)	0.164	31 (32.0)	27 (27.8)	0.090
Bilateral procedure, *yes*	91 (85.0)	97 (90.7)	−0.172	88 (90.7)	88 (90.7)	0.000
Propensity score	0.513 ± 0.082	0.487 ± 0.076	0.320	0.498 ± 0.070	0.494 ± 0.071	0.060

Data are presented as mean ± SD or n (%), as appropriate; both age and the propensity score were normally distributed in the matched groups (Lilliefors *P* = .12 to.80). The propensity score was estimated by logistic regression using the 4 covariates listed above. Matching was performed 1:1 without replacement within a caliper of 0.2 SD of the logit of the propensity score, specified a priori (caliper 0.066 on the logit scale). An absolute SMD below 0.10 was defined as adequate balance and was achieved for every covariate and for the propensity score itself. Ninety-seven of 107 patients in each group were matched; 10 patients per group remained unmatched and were excluded from the matched analysis. The region of common support was 0.358 to 0.679 in the neuro monitoring group and 0.355 to 0.680 in the non-neuro monitoring group after matching, compared with 0.358 to 0.759 and 0.343 to 0.698 before matching. Preoperative calcium was not included in the propensity score model because it showed no association with the primary outcome; its standardized mean difference was 0.315 before and 0.307 after matching, and this residual imbalance is therefore not addressed by the matched analysis.

IONM = intraoperative neuromonitoring, SD = standard deviation, SMD = standardized mean difference.

**Figure 1.**
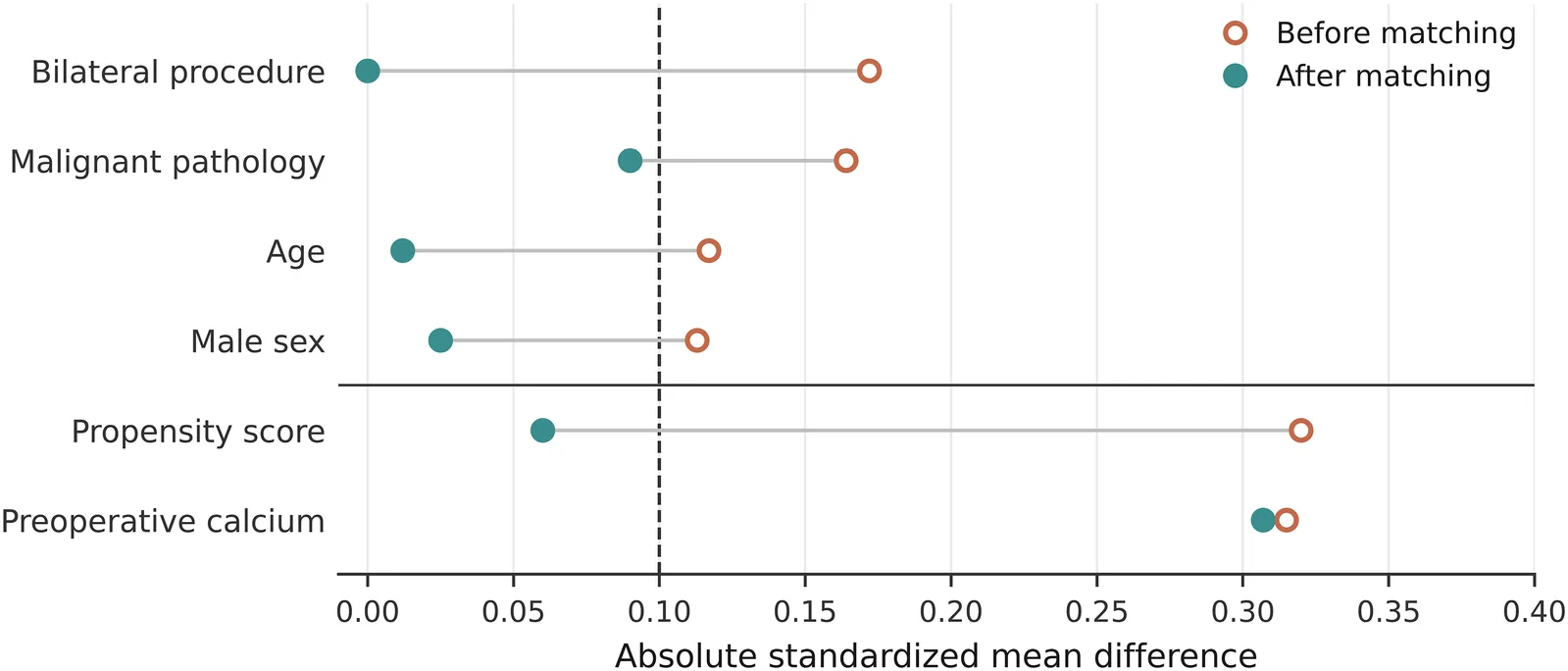
Covariate balance before and after 1:1 propensity score matching. Absolute standardized mean differences are shown for each covariate before matching (open circles) and after matching (filled circles), connected by a horizontal line. The dashed vertical line at 0.10 denotes the pre specified threshold for adequate balance. The upper block contains the 4 covariates entered into the propensity score model. The lower block contains the propensity score itself, presented as a summary measure of balance, and preoperative calcium, which was not entered into the model because it showed no association with the primary outcome; its imbalance therefore persists after matching. Matching was performed 1:1 without replacement within a caliper of 0.2 SD of the logit of the propensity score, yielding 97 matched pairs. SD = standard deviation, SMD = standardized mean difference.

**Figure 2.**
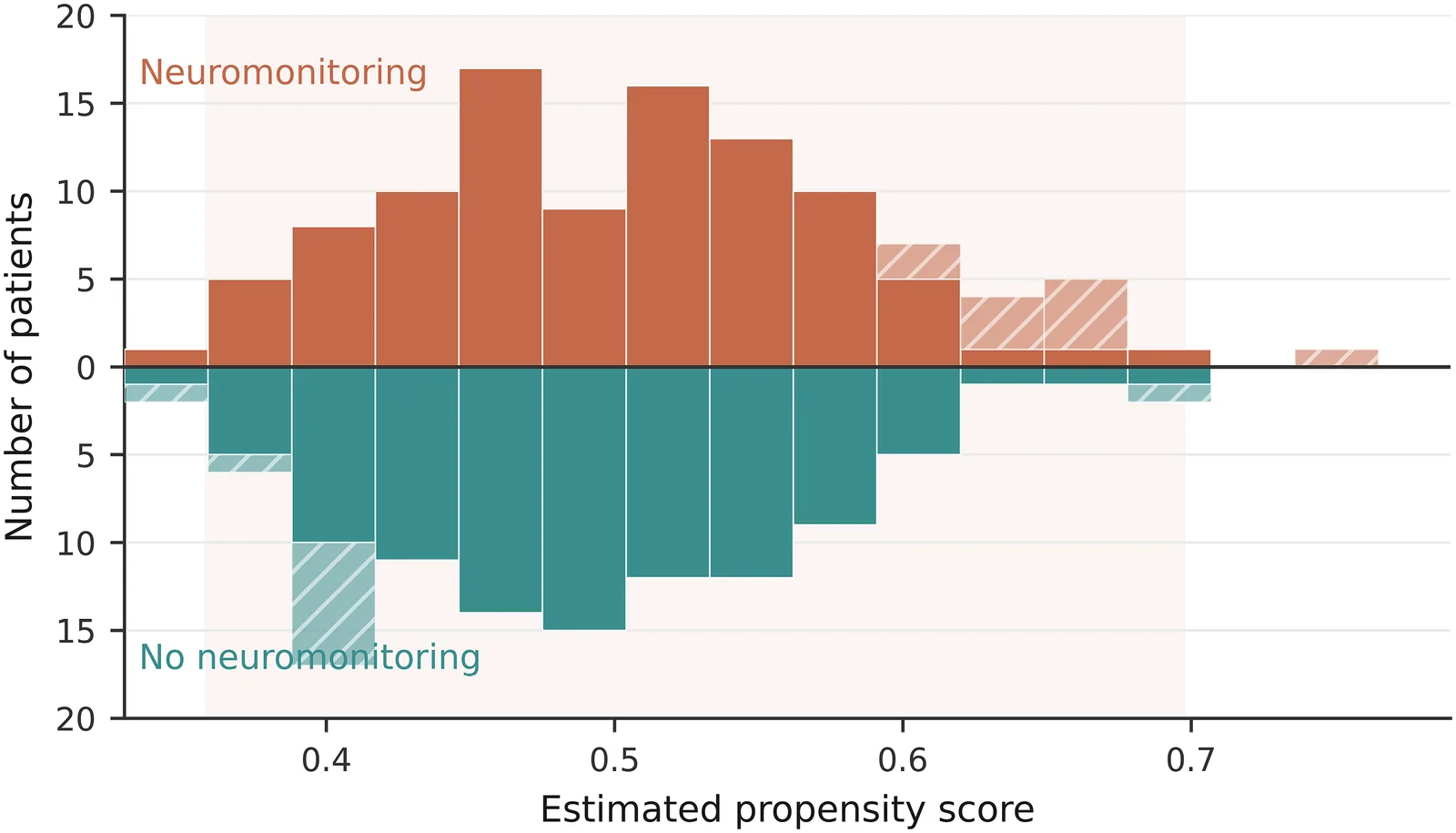
Distribution of estimated propensity scores in the neuro monitoring and non-neuro monitoring groups. Mirrored histogram of the estimated propensity score, with the neuro monitoring group plotted above the axis and the non-neuro monitoring group below. Bin width 0.029. The shaded vertical band marks the region of common support, 0.358 to 0.698. Hatched portions of each bar indicate the 10 patients per group who remained unmatched; unmatched patients in the neuro monitoring group were concentrated at the upper end of the distribution and those in the non-neuro monitoring group at the lower end. The propensity score was estimated by logistic regression using age, male sex, malignant pathology, and bilateral procedure. IONM _=_ intraoperative neuromonitoring.

In the matched cohort, any RLN palsy was recorded in 3 of 185 nerves at risk (1.6%) and 2 of 185 (1.1%), a risk difference of 0.005 (95% CI: −0.024–0.037; *P* > .99); 5 pairs were discordant, three in favor of Group 1 and two in the opposite direction, and no pair was concordant for the event. Comparable estimates were obtained when the analysis was restricted to bilateral TT (3/168 [1.8%] vs 1/162 [0.6%]; risk difference 0.012, 95% CI: −0.019–0.045; *P* = .62). Two sensitivity analyses, confined to patients whose recorded preoperative calcium was within the reference range, and to 1 record per patient, yielded risk differences of 0.006 and 0.005, and the CI included the null value in every analysis set (Table 7, Fig. 3).

**Table 7:** Recurrent laryngeal nerve palsy in the propensity score–matched cohort and in pre specified sub cohort and sensitivity analyses.

Analysis set/Outcomes	IONM, n/N (%)	Non-IONM, n/N (%)	Risk difference (95% CI)*	*P*
Propensity score–matched cohort (97 matched pairs).				
Any recurrent laryngeal nerve palsy.	3/185 (1.6)	2/185 (1.1)	0.005 (−0.024–0.037)	>.99†
Bilateral total thyroidectomy only sub cohort (84 vs 81 patients).				
Any recurrent laryngeal nerve palsy.	3/168 (1.8)	1/162 (0.6)	0.012 (−0.019–0.045)	.62
Sensitivity: patients recorded preoperative calcium within the reference range (97 vs 98 patients).				
Any recurrent laryngeal nerve palsy.	3/181 (1.7)	2/187 (1.1)	0.006 (−0.024–0.038)	.68
Sensitivity: 1 record per patient (106 vs 105 patients).				
Any recurrent laryngeal nerve palsy	3/197 (1.5)	2/201 (1.0)	0.005 (−0.022–0.035)	.68

Data are presented as n/N (%), where N is the number of nerves at risk in the corresponding analysis set. Bold *P*-values indicate statistical significance (*P* ≤ .05); no comparison reached statistical significance.

CI = confidence interval, IONM = intraoperative neuromonitoring.

* Newcombe hybrid score interval. For the matched cohort, this interval treats the 2 groups as independent and is therefore approximate; the corresponding paired estimate at the patient level was a risk difference of 0.010 with a Tango score interval of −0.046 to 0.070, which likewise includes the null value.

† The matched cohort was compared by exact McNemar test performed on the 97 matched pairs, since the number of discordant pairs was 5, well below the pre specified threshold of 25; 3 pairs were discordant in favor of the neuro monitoring group and two in the opposite direction, and no pair was concordant for the event. All remaining analysis sets were compared by Fisher exact test, applied throughout because the expected cell count was below 5 in every table. Sub cohort and sensitivity analyses constitute a separate, exploratory family and are reported without false discovery rate adjustment.

**Figure 3.**
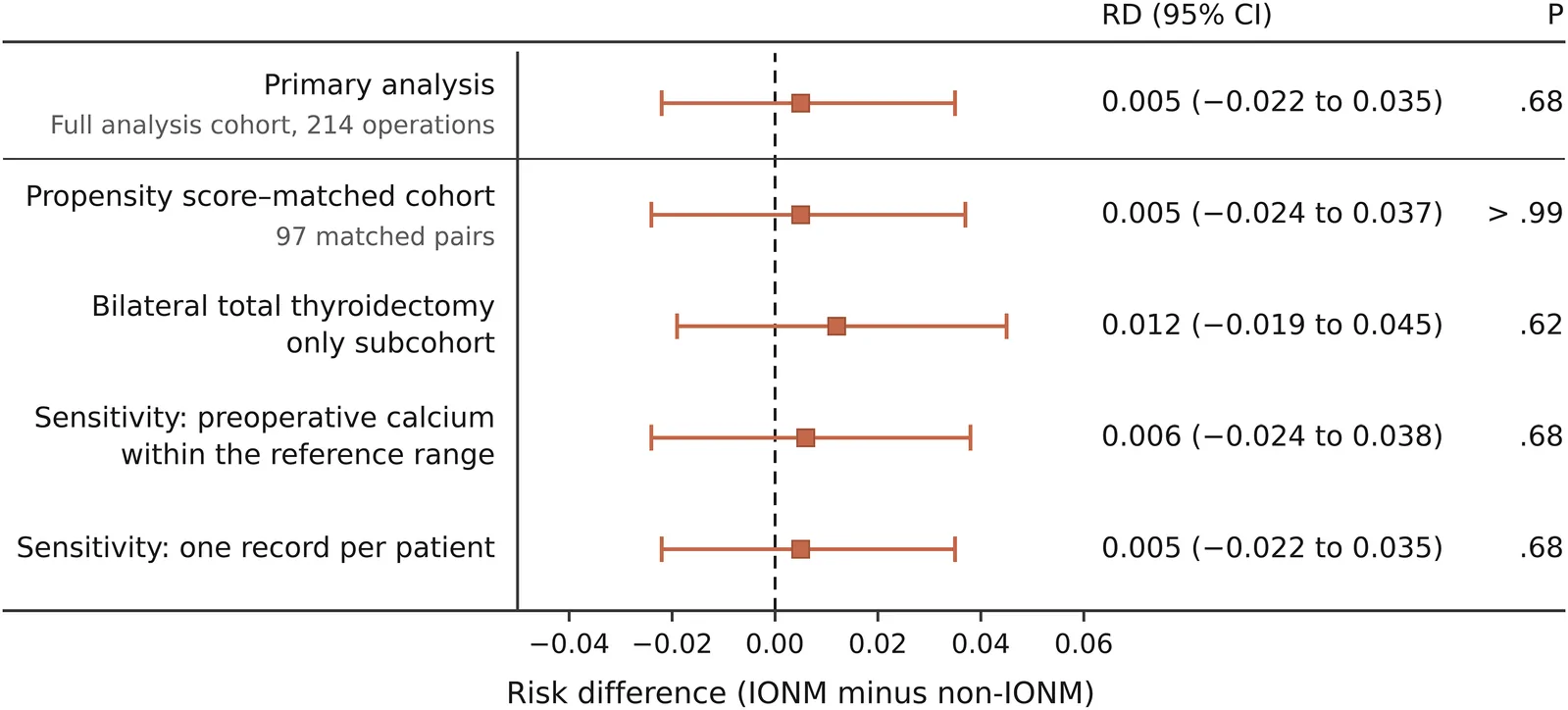
Risk difference for any recurrent laryngeal nerve palsy across the primary analysis and all pre specified sub cohort and sensitivity analyses. Squares denote the risk difference at the nerve level, expressed as the neuro monitoring group minus the non-neuro monitoring group, and horizontal lines denote the 95% Newcombe hybrid score interval. The dashed vertical line marks the null value of zero. The primary analysis is separated from the remaining analysis sets by a horizontal rule. Numerical values are those reported in Tables 3 and 7. This figure was not pre specified in the analysis plan and is presented as a post hoc graphical summary of analyses already reported in tabular form. CI = confidence interval; IONM = intraoperative neuromonitoring.

## 4. Discussion

In this retrospective cohort study of 214 thyroidectomy operations, routine IONM was not associated with a statistically significant reduction in RLN palsy compared with conventional direct visualization. At the nerve level, any RLN palsy occurred in 3 of 198 nerves (1.5%) in the IONM group and 2 of 204 nerves (1.0%) in the non-IONM group (risk difference, 0.005; 95% CI, −0.022–0.035; *P* = .68). Similarly, no significant differences were observed for transient or permanent RLN palsy. The absence of a significant association remained consistent across the propensity score–matched cohort and the pre specified bilateral TT, recorded preoperative calcium within the reference range, and 1-record-per-patient sensitivity analyses. Although preoperative calcium levels were significantly higher in the IONM group (median, 9.70 vs 9.50 mg/dL; *P* = .002), postoperative calcium and PTH levels, as well as early, transient, and permanent hypocalcemia and hypoparathyroidism, did not differ significantly between groups. These findings suggest that, within this cohort, routine IONM did not demonstrate a measurable reduction in RLN palsy or postoperative calcium–parathyroid complications compared with conventional direct visualization.

Our results suggest that the routine use of IONM during thyroid surgery does not offer a significant advantage over the conventional direct visualization technique in preventing postoperative complications. This finding, coupled with the cost of IONM, may encourage surgeons to use IONM more selectively, leading to a cost-effective approach to minimizing potential complications.

The overall incidence of RLN injury reported in the literature varies widely (0.5%–20%). Recent systematic reviews report transient and permanent RLN injury incidences of 0.6%–9.6% and 0.0%–2.0%, respectively.^[20,21]^ This wide range is likely a result of differing methodologies, including systematic postoperative laryngoscopy versus clinical dysphonia assessment. Postoperative vocal cord function was assessed by flexible fiber optic laryngoscopy, although examination findings could be retrieved only for patients in whom vocal cord dysfunction was identified. At the nerve level, transient RLN palsy occurred in 1.5% of nerves in the IONM group and 0.5% in the non-IONM group, whereas permanent palsy occurred in 0% and 0.5%, respectively; neither difference was statistically significant.

Although the relationship between IONM use and reduced RLN injury remains unclear in the literature, no permanent RLN palsy was recorded in the neuro monitoring group, and one was recorded in the comparison group. With a single event in the entire cohort, this difference cannot be distinguished from chance, and no adjusted estimate could be obtained; the observation is reported descriptively, and no protective effect is inferred. In a systematic review and meta-analysis of 164 studies, Cozzi et al also reported that IONM had no significant impact on the rate of transient or permanent RLN injury following thyroidectomy. They recommended its use in select cases, particularly to prevent bilateral vocal cord paralysis.^[20]^ Ojha et al found that while neuro monitoring may aid in identifying the RLN during thyroid surgeries, it does not reduce the risk of nerve injury compared to visual identification alone.^[22]^ In a prospective, randomized study of 1000 patients, Barczynski et al found that transient RLN palsy rates were significantly lower in surgeries using IONM: 2.9% in high-risk and 0.9% in low-risk patients.^[23]^ In their study of 6942 patients undergoing thyroidectomy for neoplastic disease, Duong et al found that IONM was associated with approximately an 80% decreased odds of severe RLN injury.^[24]^

The principal rationale for neuro monitoring advanced by surgical societies is not the reduction of endocrine complications but the management of the RLN itself, in particular the prevention of bilateral vocal cord paralysis through staging of bilateral surgery after loss of signal.^[13]^ No bilateral vocal cord paralysis occurred in either group of the present cohort, and the study is therefore uninformative for this outcome; the absence of a difference in unilateral palsy should not be read as evidence against the use of neuro monitoring for that purpose. Neuro monitoring is an adjunct to, not a substitute for, meticulous nerve identification, and no inference regarding surgical vigilance is drawn from the present findings.

In our study, transient hypocalcemia was observed in 19 patients (17.8%) in the IONM group and 30 patients (28.0%) in the non-IONM group. Permanent hypocalcemia occurred in 5 patients (4.7%) in the IONM group and 3 patients (2.8%) in the non-IONM group. The difference in transient hypocalcemia did not persist after adjustment for multiple comparisons, and operative duration was not recorded in this cohort. No mechanistic interpretation of this difference is therefore offered. Few studies have previously examined the effect of IONM on postoperative hypocalcemia. In contrast, in a retrospective study of 191 patients undergoing thyroid surgery, Demiryas et al reported significantly lower rates of temporary and permanent hypocalcemia in the IONM group.^[10]^ In a large cohort of 44,265 patients undergoing thyroidectomy, Hearn et al reported that IONM was not associated with postoperative hypocalcemia (adjusted odds ratio, 0.99; 95% CI, 0.99–1.00).^[21]^

In our study, transient hypoparathyroidism was observed in 6 patients (5.6%) in the IONM group and 9 patients (8.4%) in the non-IONM group. Permanent hypoparathyroidism developed in 2 patients (1.9%) in the IONM group, whereas none was observed in the non-IONM group. Few studies have investigated the effect of IONM on postoperative hypoparathyroidism. In a prospective study of 64 patients undergoing bilateral TT, Keklikkiran et al reported that IONM reduced the risk of hypoparathyroidism regardless of the surgeon’s experience.^[25]^ However, the present study did not demonstrate a significant association between IONM use and postoperative hypoparathyroidism, and the observed differences should therefore be interpreted descriptively.

Strengths include a relatively robust sample for a single center, and management within a single surgical unit using a uniform operative technique throughout the study period. Several limitations should be considered. Exposure was not randomly assigned but determined by calendar period, and the 2 groups did not overlap in time; the last operation without neuro monitoring preceded the first operation with neuro monitoring. Because exposure was determined by the calendar period in which surgery was performed, the effect of neuro monitoring cannot be separated from that of period, and neither matching nor restriction removes this confounding. Five nerve palsies occurred across 402 nerves at risk, so no adjusted estimate could be obtained for the primary outcome. Given the observed baseline risk of 0.98%, the smallest risk detectable with 80% power was 6.00%, and the power to detect the complete elimination of nerve palsy was 0.016; the study, therefore, cannot exclude clinically important differences in either direction. The side of nerve injury was not documented, and laryngeal examination findings were recorded only for patients in whom palsy was identified. Although ASA class was applied as an eligibility criterion, it was not available as a variable in the analysis dataset; comorbidity, operative duration, and preoperative PTH were likewise unavailable, and these variables could not, therefore, be considered as confounders. Calcium concentrations were supplied in albumin-corrected form, so the correction could not be verified independently. Preoperative calcium remained imbalanced after matching and was not entered into the model, as it showed no association with the primary outcome. The study is single-center and retrospective, and the findings should be regarded as hypothesis-generating.

In this single-center retrospective cohort, complication rates after thyroidectomy were similar with and without routine IONM, and this similarity persisted across propensity score matching and all pre specified sub cohort and sensitivity analyses. The study was not designed and is not powered to exclude clinically important differences in nerve-related outcomes. Although transient hypocalcemia occurred numerically less often in the IONM group, this difference was not statistically significant. The single permanent RLN palsy observed in the cohort precludes meaningful inference regarding a potential effect of IONM on permanent RLN palsy. These findings reinforce that meticulous technique and detailed anatomical knowledge remain the cornerstone of safe thyroidectomy; IONM is best regarded as a valuable adjunct, particularly in high-risk or complex cases, rather than a substitute for surgical expertise.

## Acknowledgments

The authors thank all the reviewers for their constructive comments that helped to improve the manuscript. This research received no funding. The authors of this work have nothing to disclose. AI-assisted tools were used solely for language editing of the manuscript; no content, data, or interpretation was generated by such tools. The authors reviewed and approved all edited text and take full responsibility for the accuracy, integrity, and originality of the content.

## Author contributions

**Conceptualization:** Huseyin Emre Arslan, Bercis Imge Ucar, Sezgin Zeren, Ali Cihat Yildirim, Ozlem Arik, Mustafa Cem Algin.

**Formal analysis:** Huseyin Emre Arslan, Bercis Imge Ucar, Ali Cihat Yildirim, Ozlem Arik.

**Investigation:** Huseyin Emre Arslan, Bercis Imge Ucar, Sezgin Zeren, Ali Cihat Yildirim, Ozlem Arik, Mustafa Cem Algin.

**Visualization:** Huseyin Emre Arslan, Mehmet Fatih Ekici.

**Methodology:** Bercis Imge Ucar, Sezgin Zeren, Ali Cihat Yildirim, Ozlem Arik, Mustafa Cem Algin.

**Data curation:** Sezgin Zeren, Mustafa Cem Algin.

**Project administration:** Sezgin Zeren, Mustafa Cem Algin.

**Supervision:** Mehmet Fatih Ekici.

**Writing – original draft:** Huseyin Emre Arslan, Sezgin Zeren, Mustafa Cem Algin.

**Writing – review & editing:** Bercis Imge Ucar, Ali Cihat Yildirim, Mehmet Fatih Ekici, Ozlem Arik.




